# Pelvic vein embolization using different embolic agents for the treatment of pelvic venous insufficiency: A systematic review and meta-analysis

**DOI:** 10.1016/j.jvsv.2026.102599

**Published:** 2026-08-09

**Authors:** Felix Strobl, Agnes Klara Böhm, Lorenz Mertens, Maximilian de Bucourt, Jan Paul Frese, Houman Jalaie, Andreas Greiner, Leon Bruder

**Affiliations:** a Department of Vascular Surgery, Charité – Universitätsmedizin Berlin, Corporate Member of Freie Universität Berlin and Humboldt-Universität zu Berlin, Berlin, Germany; b Department of Surgery, Experimental Surgery, Charité – Universitätsmedizin Berlin, Corporate Member of Freie Universität Berlin and Humboldt-Universität zu Berlin, Berlin, Germany; c Department of Radiology, Charité – Universitätsmedizin Berlin, Corporate Member of Freie Universität Berlin and Humboldt-Universität zu Berlin, Berlin, Germany

**Keywords:** Pelvic congestion syndrome, Pelvic venous disorder, Venous insufficiency, Chronic pelvic pain, Embolization

## Abstract

**Objective:**

Embolization of insufficient pelvic veins has been established as the standard first-line therapy in women with chronic pain due to pelvic venous disorder; however, the clinically available embolic devices vary widely, with coils, vascular plugs, glue, and sclerosants or combinations of these devices being the most common. This study aims to evaluate the ability of different devices to reduce pain and their individual rates of success and complications.

**Methods:**

A systematic search was conducted using PubMed, ScienceDirect, Cochrane, and ClinicalTrials.gov for studies published on the embolization of insufficient pelvic veins up until November 2025. The primary outcome measure was the effect of embolization on pelvic pain as measured using visual analog scale (VAS). The secondary end points were the technical and clinical success rates as well as the recurrence, complication, and reintervention rates.

**Results:**

A total of 28 studies met the inclusion criteria, totaling 2688 patients across 34 individual cohorts who were grouped into five treatment categories. The technical success rate was high across all the embolization methods, ranging from 98.6% to 100%, and the clinical success rate ranged from 68.3% to 100%. All the endovascular treatment options led to a reduction in pain as measured using VAS, which ranged from 4.67 (95% confidence interval [CI], 3.87-5.47) points reduction in the sclerosant-only group to 6.12 (95% CI, 5.46-6.78) in the vascular plugs group. The clinical success rate was the highest in the sclerosant-only and plugs groups at 97.5% (95% CI, 93.7-99.3) and 94.7% (95% CI, 86.9-98.5), respectively. Complications, especially in the form of device migration, most frequently appeared in the coils group at 2.63% (95% CI, 1.76-3.78) and least frequently in the sclerosant-only group at 0.45% (95% CI, 0.01-2.48). Sclerosants led to the lowest recurrence at 1.69% (95% CI, 0.49-4.87) but the highest number of reinterventions at 13.3% (95% CI, 5.1-26.8). Vascular plugs resulted in few complications (0.6% [95% CI, 0.07-2.15]), as well as low recurrence (3.47% [95% CI, 1.6-6.49]) and reintervention (2.59% [95% CI, 1.12-5.04]).

**Conclusions:**

All the embolization methods showed excellent technical success rates and marked reduction in pain as measured using VAS. Using coils seems to be associated with the highest occurrence of device migration. The vascular plugs and sclerosant-only groups had high clinical success with low complication rates. Due to the small sample sizes and lack of inter-group comparisons, the conclusions should be viewed cautiously.

**Article Highlights**

• **Type of Research:** A systematic review and meta-analysis
• **Key Findings:** A total of 2688 patients across 28 studies were pooled into five treatment groups. All the embolic agents have high technical success rates. Vascular plugs provide the largest reduction in pain levels. Sclerosants exhibit the highest clinical success rates and the lowest recurrence and migration rates.
• **Take Home Message:** All embolization methods are effective in reducing pain levels, however coil use results in highest incidence of device migration and lowest clinical success rates.

Chronic pelvic pain (CPP) is defined as acyclical pain lasting >6 months; regarding its global lifetime incidence, CPP affects an estimated 25% of women.^1^ The underlying pathologies include endometriosis, benign uterine neoplasms, malignancy, and inflammatory bowel disease, but also vascular pathologies.^2^ In up to 40% of patients, no clear pathophysiological corelate can be found.^3^ Between 16% and 31% of CPP cases appear to be linked to pelvic venous insufficiency,^2^ a manifestation of pelvic venous disorders (PeVD). The condition, formerly also described as pelvic congestion syndrome, affects predominantly parous women between the ages of 20 and 45 years, and it is characterized by reflux in pelvic veins that causes pelvic pain but can additionally manifest as dysmenorrhea, dyspareunia, urinary symptoms, or extra-pelvic varicosis of the lower body.^4^^,^^5^

Although the broader category of PeVD includes multietiological pelvic varicosis caused by venous outflow obstruction in the form of a post-thrombotic complication or obstructive syndromes, including left iliac vein compression through the iliac artery (May-Thurner syndrome) or left renal vein compression (Nutcracker syndrome),^4^ the pathomechanism of nonobstructive PeVD has been hypothesized to involve increased levels of progesterone and estrogen, especially in the context of pregnancy, where it may be aggravated by the mechanical venous outflow obstruction from the gravid uterus.^6^^,^^7^

The vessels most frequently affected by reflux are the ovarian and internal iliac veins, as well as their extensive and interconnected network of tributaries. The reliable diagnosis and categorization of PeVD remain challenging. Transabdominal or transvaginal doppler ultrasound scans are employed as the first-line diagnostic modalities upon clinical suspicion in routine practice.^8^ Enlarged pelvic veins are usually considered pathological at >5-mm diameter; however, currently, no widely accepted diameter criterion is available. Recent studies have suggested that the reflux duration might be a more reliable parameter in differentiating between PeVD as a cause of CPP and physiological variants.^8^, ^9^, ^10^ Phlebography is used to confirm the diagnosis, characterize, and further classify the findings according to the Symptoms-Varices-Pathophysiology instrument.^4^^,^^8^ Intravascular ultrasound scans have additionally been increasingly used to differentiate between obstructive and nonobstructive cases.^4^^,^^11^

The initial therapeutic approaches have focused on medical treatment with progesterone derivatives; however, these approaches result in marginal symptomatic relief.^6^ Historically employed surgical treatment options, which include hysterectomy with or without bilateral adnexectomy, have mostly been discontinued due to their highly invasive nature.^7^

Interventional treatment of PeVD is dependent on the nature of the underlying condition. For patients with primarily pelvic outflow obstruction, stenting of the iliac or renal veins has been shown to yield high rates of symptomatic relief.^12^^,^^13^ In primarily insufficient pelvic veins, embolization of the refluxing gonadal and/or iliac veins and respective tributaries has been established as the recommended therapy of choice.^8^ However, the rates of clinical success—measured by the reduction or relief of symptoms after the procedure—vary widely across studies, ranging from approximately 70% up to 100%.^14^^,^^15^

Interventional therapies of pelvic reflux have evolved from almost exclusively employing platinum or steel coils to a more varied approach using n-butyl cyanoacrylate (NBCA) glue, liquid or foam sclerosants, vascular plugs, and their combinations. Within the last years, a multitude of case-control and small randomized controlled studies have attempted to compare different interventional modalities; however, their specific setups vary widely.^16^, ^17^, ^18^, ^19^, ^20^, ^21^, ^22^ A recently published practice guideline by multiple French radiological societies recommends using whichever embolization method the performing team is the most accustomed to due to their comparably positive results; however, currently, no clear data-derived evidence exists to support this claim.^8^

The consensus on the reported items focuses on the characterization and quantification of pain-related parameters—usually assessed using the visual analog scale (VAS)—as well as dyspareunia, dysmenorrhea, increased urinary frequency, and the use of pain medication before and after intervention.^23^

This study aims to evaluate how the use of varying embolic agents in ovarian or iliac vein embolization affects the ability of the procedure to reduce pain and improve subjective outcome measures while also examining device-specific complications and recurrence rates.

## Methods

This systematic review and meta-analysis was conducted according to Preferred Reporting Items for Systematic Reviews and Meta-Analyses guidelines. It has been registered on PROSPERO, with the reference number CRD420251240231. Formal approval by the local institutional ethics committee was not necessary, as this study solely compiles previously published data collected from electronic databases.

### Search strategy

**Fig 1:**
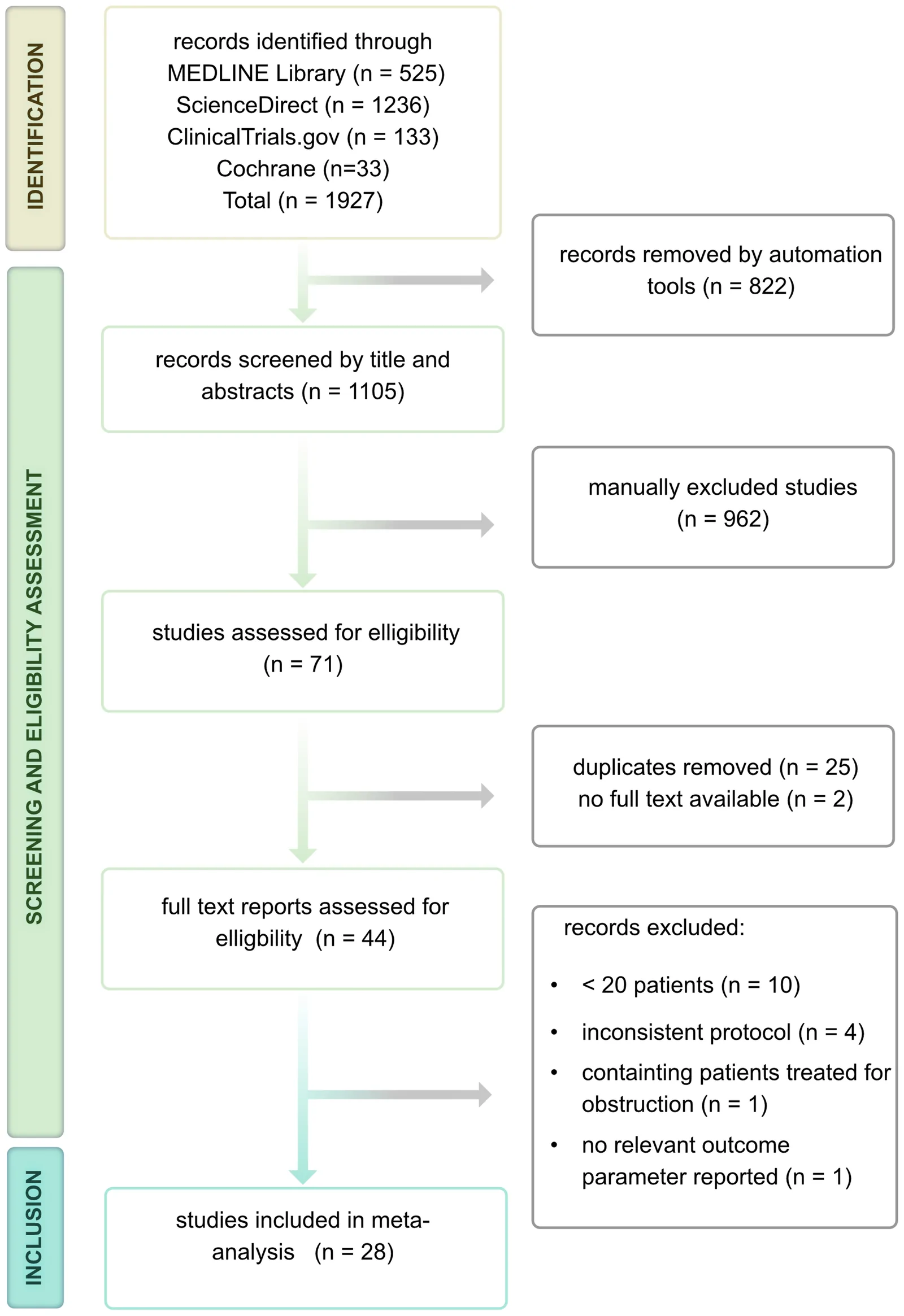
Flowchart of the data extraction process.

Due to the inconsistent nomenclature (pelvic congestion, PeVD, pelvic venous disease, pelvic varicosis, and pelvic venous reflux), the search was conducted using a set of wide-scale descriptors in the databases of PubMed, ScienceDirect, Cochrane Central Register of Controlled Trials, and ClinicalTrials.gov from the creation of the databases until November 2025. The specific search terms used for each database, as well as the specific yield, can be found in Supplementary Data 1 (online only). Articles published in either English, German, or Spanish were considered. The inclusion criteria were as follows: (1) intervention studies of pelvic venous insufficiency, (2) endovascular treatment, (3) ≥1 month follow-up, (4) >20 patients with a single consistent protocol or comparing two protocols with >20 patients in each arm, and (5) reporting of at least one of the main outcome parameters (change in VAS score and clinical success). The exclusion criteria were as follows: (1) absence of any one inclusion criterion, (2) additional treatment of venous obstruction (eg, stenting due to May-Thurner or Nutcracker syndrome), and (3) overlapping patient populations in multiple studies. In case only a singular cohort fulfilled all the inclusion criteria, the cohort was included if sufficiently separately characterized. Cohorts combining interventional therapy with medical treatment were excluded. Two independent reviewers (F.S. and L.B.) assessed the initial study output (Fig 1). In case of disagreement, a third independent reviewer was consulted (A.K.B.).

### Data extraction and definitions of outcome measures

Study characteristics (eg, type of study, duration, number of patients included, patients completing follow-up, and follow-up time), population characteristics at baseline, and outcome parameters of interest (ie, targeted vessels, embolic agent/device, technical and clinical success, VAS before and after intervention, occurrence of complications, recurrence, and reintervention) were extracted for every cohort. VAS reduction was always calculated with resting pain; if pain was analyzed depending on the position (eg, standing and sitting), the highest score was chosen. In case of comparative studies where multiple cohorts were treated with varying protocols and both fulfill the inclusion criteria, they were treated as separate studies. Complications were categorized into either migration/embolism or site-of-intervention complications (eg, post-embolization syndrome [PES], perforation, rupture, and device protrusion). Minor complications at access sites, short-term postprocedural pain up to 72 hours, asymptomatic thrombosis of nontarget veins, or medical complications were not analyzed.

Recurrence was defined as the recurrence of pelvic pain with or without radiographic recurrence. The solitary recurrence of lower-body varicosis was not considered. Technical success was defined as the completion of embolization and the subsequent occlusion of the intended vessels. The definition of clinical success was adapted from the included studies, which mostly depict clinical success as an improvement in subjective experience by patients (also including dysmenorrhea to dyspareunia and increased urinary frequency), although not always solely linked to the VAS reduction.

### Statistical analysis

Continuous variables are described as means and 95% confidence intervals (CIs). Categorical variables are presented as proportions (counts/100 embolizations) and 95% CIs. Random-effects meta-analyses of changes in VAS scores were performed separately for each intervention category using restricted maximum likelihood (REML) estimation for the between-study variance $\tau^{2}$. Input data consisted of mean changes and their standard deviations (SDs), with pooled estimates reported alongside 95% CIs. Study weights were derived from the inverse-variance method, and heterogeneity was quantified using $\tau^{2}$, Cochran's Q test, and the *I*^2^ statistic. Leave-one-out analyses were conducted to assess robustness. In one study,^16^ the mean and SD were estimated using the approximate Bayesian computation method described by Kwon et al^24^ from the median and 95% CI.

Complication, recurrence, and reintervention rates as well as clinical success rates are reported as counts (proportions) with 95% CI. We used a Bayesian generalized linear mixed model with the Clopper-Pearson exact method to model events/100 patients with 95% CI. Heterogeneity was estimated using the maximum-likelihood estimator for $\tau^{2}$; *I*^2^ was based on Q. *P* values were calculated using the likelihood ratio test. For the follow-up time as well as patient characteristics (age, parity, and pretreatment VAS), the grand means were calculated (weighted by the number of patients) overall as well as per group. They are reported as the overall mean (range of means), unless stated otherwise. The meta-analysis results were visualized using forest plots and diamond plots generated in R (Open Source, version 4.4.1, R Studio Version 2023.12.0), reporting the overall Z-statistic for the random-effects model as well as prediction intervals. The threshold for statistical significance was defined as *P* ≤ .05. Statistical analyses were likewise performed in R.

### Risk of bias analysis

Risk of bias (ROB) was independently assessed by two reviewers (F.S. and L.B.) using the Cochrane Collaboration assessment tool Risk of Bias In Nonrandomized Studies—of Interventions (ROBINS-I) for nonrandomized studies as well as the Risk-of-bias Tool for Randomized Trials (ROB-2) for the included randomized controlled trials. The results of individual domains as well as overall ROB were visualized for each study (Supplementary Figs 1 and 2, online only).

## Results

### Study characteristics and outcome parameters

A total of 2688 patients were included in this study, across 35 individual cohorts and from 28 individual studies.^13^^,^^14^^,^^16^, ^17^, ^18^, ^19^, ^20^, ^21^, ^22^^,^^25^, ^26^, ^27^, ^28^, ^29^, ^30^, ^31^, ^32^, ^33^, ^34^, ^35^, ^36^, ^37^, ^38^, ^39^, ^40^, ^41^, ^42^, ^43^ Data on the study type, methodology, and specific characterization are depicted alongside primary outcome measures and follow-up times in Table I. The secondary outcome measures are depicted in Table II. A mean of 79.1 (range, 21-261) patients per cohort were followed up over an overall mean period of 30.0 months (range of means, 6-58.7). Patients were all female with an overall mean age of 41.0 years (31.3-58.8), with a parity rate of 90.5% (37-100) and 2.28 parities per patient (2.0-3.0). PeVD was diagnosed using doppler ultrasound and was confirmed using venography; only two studies opted for additional intravenous ultrasound scans to further classify pathology.^13^^,^^40^ Cohorts were pooled according to the embolic device or agent used (Graphical Abstract). The glue group (GG) included five cohorts, all using NBCA glue combined with Lipiodol, and a total of 272 patients with a mean age of 37.1 (31.3-41.7) years who were followed up over an average of 12.1 (6-24.5) months. The coils group (CG) consisted of 11 cohorts employing mostly fibered platinum coils in a total of 1064 patients with a mean age of 40.3 (31.3-43.7) years who were followed up for a mean of 30.1 (1-58.7) months. The plugs group (PG) consisted of three cohorts and a total of 334 patients treated with Amplatzer II or IV plugs at a mean age of 44.1 (44.3-47.1) years who were followed up over a mean period of 48.2 (12-58.7) months. The sclerosant group (SG) included 222 patients across five cohorts who were treated with either liquid (Onyx) or one of various foam sclerosants (sodium-tetradecyl-sulfate, ethanolamine, and polidocanol). Patients had a mean age of 38.0 (33.8-38) years and were followed up for 35.0 (12-99) months. The coils plus sclerosant group (CSG) included a total of 683 patients across eight cohorts, combining mostly fibered platinum coils with a variety of liquid or foam sclerosants. The patient age was 40.8 (32.3-58.7) years in this group, and the follow-up time was 25.2 (6-45) months. Other combinations of embolic devices and agents included plugs plus sclerosant (one cohort, 25 patients) and coils plus glue (one cohort, 88 patients). These groups were excluded from the meta-analysis due to being limited to one cohort each and the overall small sample sizes. Preintervention VAS scores varied across the groups and ranged from 6.35 (SD, 1.96) in the sclerosant-only group to 7.57 (SD, 1.29) in the coils plus sclerosants group (Table I).

**Table I:** Study characteristics and primary outcome parameters

Study	Country	N =	Device/Agent specification	Control group specification	Target vessels	Study type	Age, years	VAS before	VAS after	Follow-up, month
Glue										
Maleux et al^26^	Belgium	41	NBCA + Lipidol		OV uni/bilateral	Prospective	37.8			19.9 (range 1-61)
Van der Vleuten et al^32^	Netherlands	21	NBCA + Lipidol		OV uni/bilateral	Retrospective	41.7			18.1 ± 11.6
Leal-Lorenzo et al^39^	Spain	30	NBCA + Lipidol		OV bilateral	Prospective	32.3	7.74 ± 1.03	2.16 ± 1.86	24.5 ± 6.5
Zaghloul et al^21^ (Glue cohort)	Egypt	80	NBCA + Lipidol		OV uni/bilateral	Retrospective	33.6	7.21 ± 1.34	1.7 ± 1.32	6
Hipola et al^43^	Spain	100	NBCA + Lipidol		OV uni/bilateral	Retrospective	40.2	7.74 ± 1.03	1.77 ± 1.31	12
Overall		272					37.1	7.54 ± 1.15	1.80 ± 1.39	12.1 ± 7.3
Coils										
Chung and Huh^28^	South Korea	52	unspecified	vs Hysterectomy ± Oophorectomy	OV uni/bilateral	RCT	40.1	7.8 ± 1.2	3.2 ± 0.9	26.5 ± 5.2
Kwon et al^30^	South Korea	67	unspecified		OV unilateral	Retrospective	39.1			44.8 ± 21
Laborda et al^33^	Spain	202	Steel or fibered platinum coils		OV + IIV bilateral	Prospective	43.5	7.34 ± 0.7	0.78 ± 1.2	up to 60
Nasser et al^14^	Brazil	113	Fibered steel coils		OV bilateral	Retrospective + Prospective follow-up	43.7	7.34 ± 0.071	0.47 ± 0.050	12
Edo Prades et al^35^	Spain	22	Fibered platinum coils		OV/IIV unilateral	Retrospective	42.6			50 ± 30 (range 2-90)
Siqueira et al^36^	Brazil	22	Fibered platinum coils		OV ± IIV uni/bilateral	Retrospective	38.4	8.4 ± 1.6	5.2 ± 3.2	10.2 ± 7.9 (range 3-24)
Guirola et al^16^ (Coil cohort)	Spain	50	Fibered platinum coils	vs Amplatzer Plug II	OV + IIV bilateral	RCT	41	median 7 (CI, 7, 8)	median 1,4 (CI, 0.85, 1,91)	12
De Gregorio et al^17^ (Coil cohort)	Spain	261	Fibered platinum coils	vs Amplatzer Plug II/IV	OV + IIV bilateral	Retrospective	44.7	6.9 ± 2.0	2.7 ± 2.0	58.7 ± 5.7 (range 36-60)
Gavrilov et al^37^	Russia	67	Fibered nickel-chromium coils	vs Endoscopic Resection	OV uni/bilateral	Retrospective	31.3	7.5 ± 0.2	0.5 ± 0.2	36
Gavrilov et al^25^	Russia	177	Steel or fibered nickel-chromium coils	vs Endoscopic Resection	OV uni/bilateral	Retrospective, Multi-Center	32.1	6.3 ± 1.9	2.1 ± 1.4	60
Yesiltas et al^22^	Turkey	31	Fibered platinum coils	vs Fibered platinum coils + Onyx	OV uni/bilateral	Retrospective	37.6	9.23 ± 0.67	3.61 ± 1.58	12
Overall		1064					40.3	7.33 ± 1.24	1.79 ± 1.30	30.1 ± 24.6
Plugs										
Guirola et al^16^ (Plug cohort)	Spain	50	Amplatzer Plug II	vs Coils	OV + IIV bilateral	RCT	44.3	median 8 (CI, 7, 8)	median 1,1 (CI, 0.55, 1,64)	12
De Gregorio et al^17^ (Plug cohort)	Spain	259	Amplatzer Plug II/IV	vs Coils	OV + IIV bilateral	Retrospective	44.7	7 ± 0.0	1.5 ± 1.2	58.7 ± 5.7 (range 36-60)
De Gregorio et al^18^ (Plug only cohort)	Spain	25	Amplatzer Plug II	vs Plugs + Sclerosant	OV + IV bilateral	Prospective, Multi-Center	47.1	7.8 ± 0.8	1.3 ± 0.8	12
Overall		334					44.8	7.14 ± 0.33	1.43 ± 1.28	48.2 ± 20.2
Sclerosant										
Gandini et al^31^	Italy	38	Sodium-tetradecyl-sulfate (foam)		OV uni/bilateral	Retrospective	36.9	7.8 ± 1.8	2.7 ± 2.8	12
Gandini et al^34^	Italy	26	Sodium-tetradecyl-sulfate (foam)		OV uni/bilateral	Retrospective	37.3	7.5 ± 1.4	2.8 ± 0.7	12
Jambon et al^38^	France	73	Onyx (Liquid)		OV + IIV bilateral	Prospective	41	6.07 ± 2.79	1.01 ± 1.82	28 (Q1-Q3: 24.0-29.2)
Shahat et al^40^	Egypt	40	Ethanolamine (foam)		OV ± IIV unilateral	Retrospective	33.8	4.45 ± 1.46	1.30 ± 0.13	12
Rossi et al^13^	Brazil	45	Polidocanol (foam)		OV uni/bilateral	Retrospective + Prospective follow-up	38	8.5 ± 1.5	3.1 ± 1.1	99 ± 24
Overall		222					38.0	6.35 ± 1.96	1.99 ± 1.41	34.9 ± 42.2
Coils + Sclerosant										
Venbrux et al^27^	United States	56	Fibered platinum coils Gelfoam/sodium morrhuate		OV bilateral	Prospective	32.3	7.8 (range, 3.2-9.8)	2.7 (range, 0.0-6.9)	22.1 (range 6-38)
Kim et al^29^	United States	127	Steel or platinum coils Gelfoam/sodium morrhuate		OV + IIV bilateral	Retrospective + Prospective follow-up	34	7.6 ± 1.8	2.9 ± 2.8	45 ± 18
Chen et al^19^ (proximal cohort)	China	94	Fibered platinum coils Polidocanol		OV ± IIV uni/bilateral	Retrospective, Multi-Center	40	7 (5-9)		12
Zhou et al^20^ (Sclerosant cohort)	China	77	unspecified Polidocanol		OV uni/bilateral	Propensity Score Matched	58.7	6.23 ± 0.43	2.14 ± 0.35	36
Liang et al^41^	Australia	139	Fibered platinum coils Sodium-tetradecyl-sulfate/polidocanol		OV ± IIV uni/bilateral	Retrospective	47	7.8 ± 1.7	2.7 ± 1.7	35 (range 12-60)
Yesiltas et al^22^	Turkey	59	Fibered platinum coils Onyx		OV uni/bilateral	Retrospective	40.4	9.17 ± 0.77	2.51 ± 2.13	12
Zaghloul et al^21^ (Sclerosant cohort)	Egypt	87	Fibered platinum coils Polidocanol		OV uni/bilateral	Retrospective	34.2	7.24 ± 1.23	1.14 ± 0.904	6
Wang et al^42^	China	44	unspecified		OV bilateral	Retrospective	36.2	7.71 ± 0.84	2.93 ± 1.19	6
Overall		683					40.8	7.57 ± 1.29	2.37 ± 1.58	25.2 ± 18.8
Plug + Sclerosant										
De Gregorio et al^18^ (Plug + Sclerosant cohort)	Spain	25	Amplatzer II, Foam	vs Plugs only	OV + IIV bilateral	Prospective, Multi-Center		7.9 ± 1.3	1.3 ± 1.1	12
Coil + Glue										
Zhou et al^20^ (Glue cohort)	China	88	Coil + NBCA	Coil + Sclerosant	OV uni/bilateral	Propensity Score Matched		6.57 ± 0.79	2.05 ± 0.37	36

*CI*, confidence interval; *IIV*, Internal iliac vein; *NBCA*, N-butyl cyanoacrylate; *OV*, ovarian vein; *RCT*, randomized controlled trial; *SD*, standard deviation; *VAS*, visual analog scale.

Data are presented as mean ± SD unless stated otherwise. Grand means are presented in bold lettering.

**Table II:** Secondary outcome parameters, sorted by embolization method

Study	Technical success	Clinical success	Complications	Recurrence evaluation	Recurrence	Re-intervention
Glue						
Maleux et al^26^	40/41 (98%)	28/41 (68.3%)	Migration 2/41 (4%)	Clinical	0/41 (0%)	
Van der Vleuten et al^32^		16/21 (76.2%)				9/21 (42.9%)
Leal-Lorenzo et al^39^	29/30 (97%)					
Zaghloul et al^21^ (Glue cohort)	80/80 (100%)	75/80 (93.8%)	Migration 1/80 (1.3%)	Clinical, DUS	5/80 (6.2%)	
Hipola et al^43^	100/100 (100%)	89/100 (89%)	0/100 (0%)	Clinical	11/100 (11%)	4/100 (4%)
Overall	98.6%	86.0%			7.2%	10.7%
Coils						
Chung and Huh^28^	50/52 (96.2%)		Migration 2/50 (3.8%)			
Kwon et al^30^	67/67 (100%)	55/67 (82.1%)	Migration 2/67 (3%)			
Laborda et al^33^	202/202 (100%)	168/202 (93.9%)	Migration 4/202 (1.9%)	Clinical	7/202 (3.9%)	
Nasser et al^14^	113/113 (100%)		Migration 4/113 (3.5%)			
Edo Prades et al^35^	22/22 (100%)	16/22 (72%)	0/22 (0%)			
Siqueira et al^36^	22/22 (100%)	20/22 (90.9%)	Venous rupture 2/22 (9.1%)			4/22 (18.1%)
Guirola et al^16^ (Coil cohort)	49/50 (96%)	47/49 (95.9%)	Migration 3/50 (6%). Extravasation 6/50 (12%)			7/50 (14%)
De Gregorio et al^17^ (Coil cohort)	261/261 (100%)		Migration 10/261 (3.8%)	Clinical	17/261 (6.5%)	11/261 (4.2%)
Gavrilov et al^37^		64/67 (95.5%)	Coil protrusion 3/67 (4.5%). PES 13/67 (19.4%)	Clinical	0/67 (0%)	
Gavrilov et al	177/177 (100%)	131/177 (74%)	Migration 2/177 (1.1%). Coil protrusion 10/177 (5.6%). PES 35/177 (20%)	Clinical, DUS, Venography	29/177 (16%)	
Yesiltas et al^22^			Migration 1/31 (3.2%). Extravasation 3/31 (9.7%)			
Overall	99.4%	82.7%			7.5%	6.6%
Plugs						
Guirola et al^16^ (Plug cohort)	49/50 (96%)	48/50 (96%)	Migration 1/50 (2%). Extravasation 2/50 (4%)			2/50 (4%)
De Gregorio et al^17^ (Plug cohort)	259/259 (100%)		Migration 1/259 (0.3%)	Clinical	9/259 (3.4%)	6/259 (2.3%)
De Gregorio et al^18^ (Plug only cohort)	25/25 (100%)	23/25 (92%)	0/25 (0%)			
Overall	99.4%	94.7%			3.5%	2.6%
Sclerosant						
Gandini et al^31^	38/38 (100%)		0/38 (0%)	Clinical, DUS	0/38 (0%)	
Gandini et al^34^	26/26 (100%)		0/26 (0%)		0/26 (0%)	
Jambon et al^38^	73/73 (100%)	70/73 (95.9%)	PES 3/73 (4.1%)	Clinical	2/73 (2.7%)	
Shahat et al^40^	40/40 (100%)	39/40 (97.5%)	Migration 1/40 (2.5%)	Clinical, DUS	1/40 (2.5%)	
Rossi et al^13^		45/45 (100%)	Venous perforation 2/45 (4.4%)			6/45 (13.3%)
Overall	99.7%	97.5%			1.7%	13.3%
Coils + Sclerosant						
Venbrux et al^27^	56/56 (100%)	54/56 (96%)	Migration 2/56 (3.6%)	Clinical, Venography	3/56 (5.4%)	
Kim et al^29^	127/127 (100%)		Migration 2/127 (1.6%)	Clinical	6/127 (5%)	
Chen et al^19^ (proximal cohort)	94/94 (100%)	81/94 (86.2%)	0/94 (0%)			
Zhou et al^20^ (Sclerosant cohort)			Migration 6/77 (10.2%)	Clinical, DUS	20/77 (26%)	
Liang et al^41^		72/86 (84%)	0/139 (0%)			4/86 (4.7%)
Yesiltas et al^22^			Migration 0/59 (0%). Extravasation 4/59 (6.8%)			
Zaghloul et al^21^ (Sclerosant cohort)	87/87 (100%)	87/87 (100%)	0/87 (0%)	Clinical, DUS	0/87 (0%)	
Wang et al^42^		36/44 (81.8%)	Migration 1/44 (2.3%)			
Overall	100%	89.9%			9.1%	4.7%
Plug + Sclerosant						
De Gregorio et al^18^ (Plug + Sclerosant cohort)	25/25 (100%)	23/25 (92%)	0/50 (0%)			
Coil + Glue						
Zhou et al^20^ (Glue cohort)			Migration 2/88 (2.3%)	Clinical, DUS	11/88 (12.5%)	

*DUS*, Doppler ultrasound; *PES*, post-embolization syndrome.

Data are presented as counts (proportions).

### Risk of bias analysis

ROB analysis of the two included randomized controlled trials showed moderate bias mainly due to insufficient information on the randomization and allocation processes (Supplementary Fig 1, online only). Analysis of the nonrandomized intervention studies showed overall high ROB, with 12 studies classified as severe ROB, 12 studies as moderate, and only 2 studies as low ROB (Supplementary Fig 2, online only). Several of the included studies were conducted by the same authors.^16^, ^17^, ^18^^,^^25^^,^^31^^,^^34^^,^^37^ No evidence exists of patient populations overlapping in the information provided in the individual studies.

### Meta-analysis of reduction of pain levels

**Fig 2:**
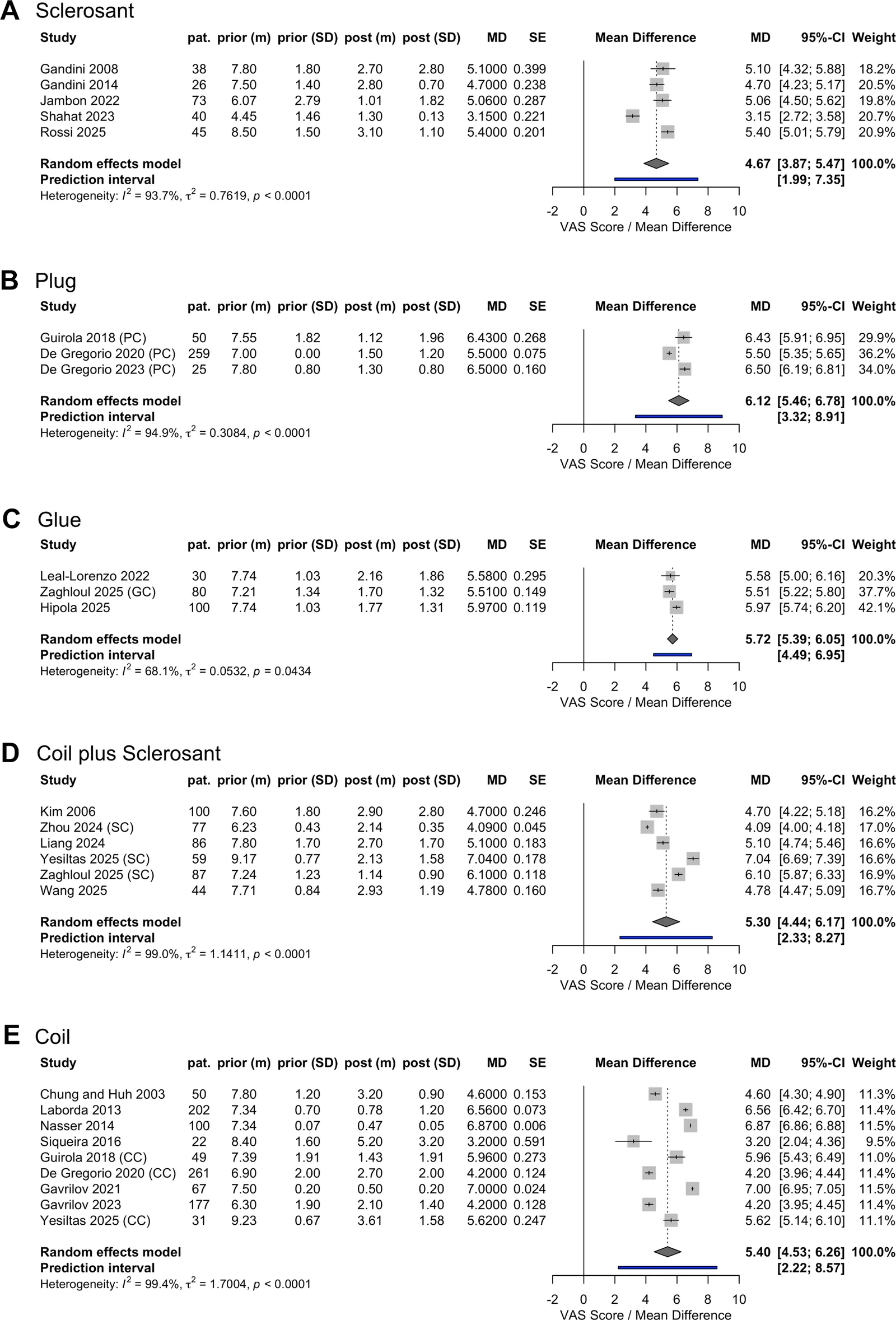
Meta-analysis of VAS score change pre- and post-embolization by method. **A,** Sclerosant only. **B,** Vascular plugs. **C,** Glue. **D,** Coils plus Sclerosant. **E,** Coils. *CI*, Confidence interval; *MD*, mean difference; *SD*, standard deviation; *SE*, standard error; *VAS*, visual analog scale.

Each embolization method led to a relevant decrease in the mean pain level measured using VAS (Fig 2). This decrease was pronounced in the PG, where the VAS score was reduced by a mean of 6.12 points (95% CI, 5.46-6.78), but with high between-study heterogeneity at *I*^2^ = 94.9% (Fig 2, *B*). The VAS score was decreased in the GG, with a mean score of 5.72 points (95% CI, 5.39-6.35), a narrower prediction interval of 4.49 to 6.95 points, and lower but still significant heterogeneity at *I*^2^ = 68.1% Fig 2, *C*). The combination of coils and a sclerosant agent decreased the VAS score by a mean of 5.30 points (95% CI, 4.44-6.17), with high interstudy heterogeneity of *I*^2^ = 99.0% (Fig 2, *D*). With the use of coils alone, the VAS score decreased to a mean of 5.40 points (95% CI, 4.53-6.26). The coils group – by far the largest in number of studies and patients included – shows the largest prediction interval (95% CI, 2.22-8.57) as well as the highest heterogeneity at *I*^2^ = 99.4% (Fig 2, *E*). VAS score reduction in the sclerosant cohort was observed with a mean of 4.67 points (95% CI, 3.87-5.47), with heterogeneity at *I*^2^ = 93.7% (Fig 2, *A*). The combination of coils with glue was only implemented in one group and led to a reduction of 4.52 points in the VAS score. Combining plugs with sclerosant was similarly only implemented in one group and led to 6.60 decrease in the VAS score (Table I).

### Meta-analysis of technical and clinical success

**Fig 3:**
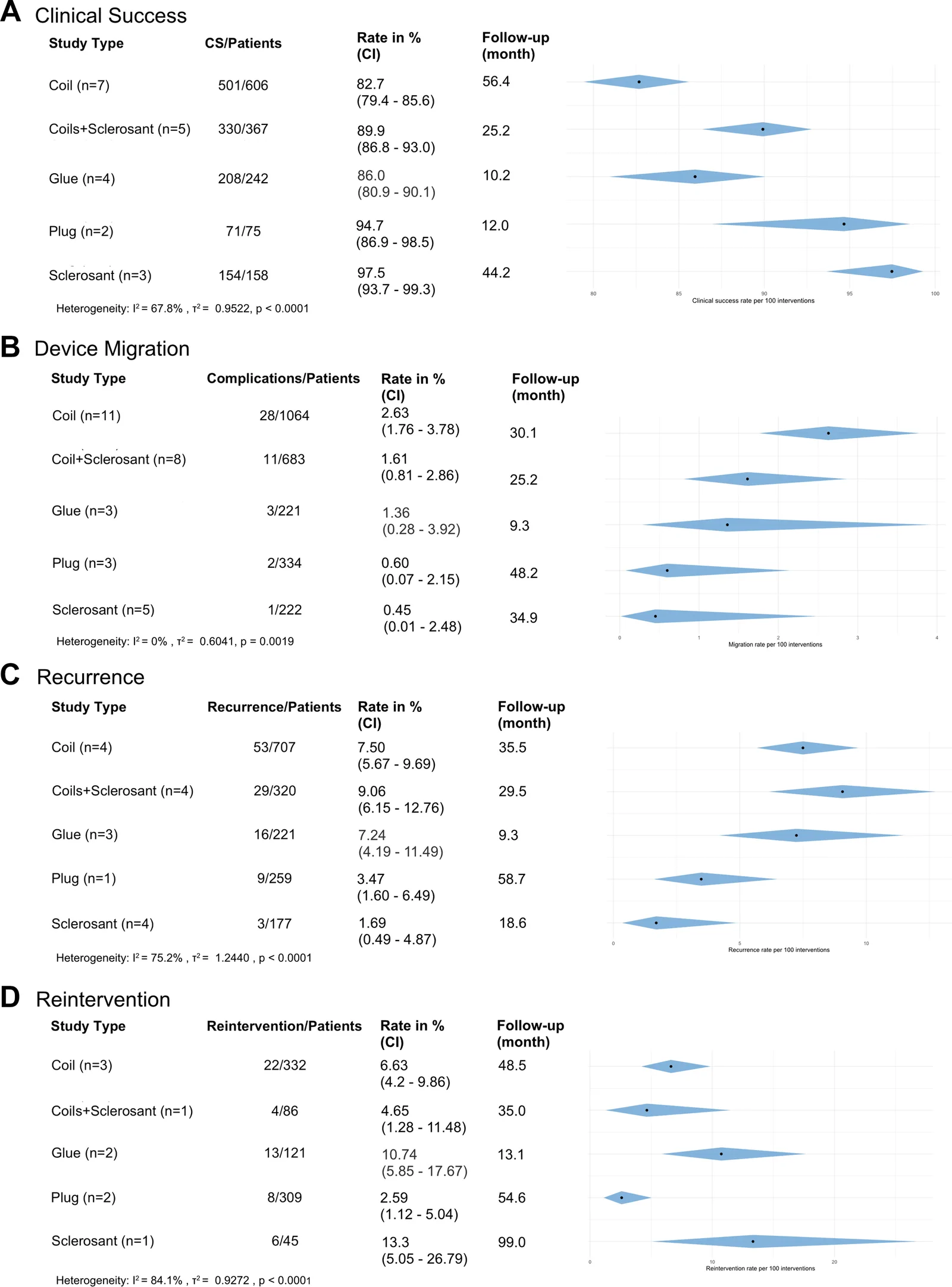
Meta-analysis of **(A)** clinical success, **(B)** complications (migration), **(C)** recurrence, and **(D)** reintervention rates by method. Follow-up is presented as the weighted mean in months. *Diamond* plots show the mean and 95% confidence interval (*CI*). *SE*, Standard error.

The technical success rate was hi[[parms resize(1),pos(50,50),size(200,200),bgcol(156)]]|PDF FILE NOT READYh across all the embolization methods, ranging from 98.6% in the GG to 100% in the CS group (Table II). The clinical success rate, as self-reported in individual studies, ranged from 97.5% (95% CI, 93.7-99.3; Fig 3, *A*) in the SG to 82.7% (95% CI, 79.4-85.6) in the CG. The clinical success rate in the PG was 94.7% (95% CI, 86.9-98.5). In the CS group, 89.9% (95% CI, 86.8-93.0) of cases were reported as clinically successful, as well as 86.0% (95% CI, 80.9-90.1) in the GG. Between-group heterogeneity was significant at *I*^2^ = 67.8%.

### Meta-analysis of the occurrence of complications, recurrence, and reintervention

The meta-analysis of complications (Fig 3, *B*) was limited to embolic agent or device migration due to limited data and the lack of the comparability of other complications. Migration of coils, either to a proximal greater vein or the pulmonary arteries, was noted in 2.63% (95% CI, 1.76-3.78) of patients in the CG and 1.61% (95% CI, 0.81-2.86) in patients treated with additional sclerosant (CSG). Migration of the injected glue (GG) occurred in 1.36% (95% CI, 0.28-3.92) of cases. Migration occurred in 0.6% (95% CI, 0.07-2.15) and 0.45% (95% CI, 0.01-2.48) of cases in the PG and SG, respectively. Within-group heterogeneity was low at *I*^2^ = 0% (95% CI, 0.0%, 40.8%).

Embolization performed with a combination of coils and glue was associated with device migration in 2.3% of cases; using plugs plus sclerosant was associated with no cases of migration (0.0%), however both of these combinations were only performed in one study each. Device migration, along with other major complications, is listed in Table II.

Recurrence of pain with or without radiologically proven recanalization of embolized vessels was noted in 9.1% (95% CI, 6.2-12.8; Fig 3, *C*) of CSG patients at the mean follow-up period of 29.5 months (range of means, 6-45), 7.5% (95% CI, 5.7-9.7) in the CG at the mean follow-up period of 35.5 months (range of means, 36-60), and 7.2% (95% CI, 4.2-11.5) in the GG at the mean follow-up period of 9.3 months (range of means, 6-19.9). In the PG, recurrence was analyzed in only one study, where the occurrence of returning symptoms was reported at 3.5% (95% CI, 1.6-6.5) at the mean follow-up period of 58.7 months. Recurrence was observed in 1.7% (95% CI, 0.5-4.9) of patients treated with sclerosant only at the mean follow-up period of 18.6 months (range of means, 12-28).

Lastly, reintervention events were analyzed across embolization modalities (Fig 3, *D*). Reintervention was observed in patients in the SG at 13.3% (95% CI, 5.05-26.8); however, data were only available from one study, with the mean follow-up time of 99 months. Reintervention was deemed necessary in 10.7% (95% CI, 5.9-17.7) of patients in the GG at the 13.1-month follow-up (range of means, 12-18.1), 6.6% (95% CI, 4.2-9.9) in the CG at 48.5 months (range of means, 10.2-58.7), and 4.7% (95% CI, 1.3-11.5) of patients in the CSG—with again only one study reporting reintervention in the latter group at the 35-month mean follow-up. When vascular plugs were employed, reintervention was noted in 2.6% (95% CI, 0.8-4.4) of patients at the mean follow-up of 54.6 months (range of means, 12-58.7).

## Discussion

To the best of our knowledge, this study presents the first comparative analysis of embolization techniques used for the treatment of insufficient pelvic veins. While initial studies on the topic have mostly compared embolization with medical or surgical treatment, the focus of many recent comparative studies has shifted toward the comparative analysis of different interventional modalities. It was therefore our aim to create a synthesis of the existing evidence to enable clinicians to choose the most suitable option.

Overall, every evaluated method led to significant reduction in pain levels. Reduction of the VAS score seemed the most effective using vascular plugs or NBCA glue. The least effect was noted in patients treated with sclerosant only. However, this group exhibited the lowest level of preintervention VAS score (Table I). This finding may also explain why the sclerosant-only group showed the highest clinical success. Most studies have evaluated clinical success on the basis of the reduction of VAS scores below a certain threshold; thus, it is more readily achievable in patients with less severe pain pre-intervention. In the VAS score analysis, all the embolic agents showed high between-study heterogeneity. Although the individual studies all showed a positive effect, slightly varying means with very narrow SDs often lead to these highly significant results. This is likely caused by the subjective and examiner-dependent nature of pain. It is also rarely specified in which body position the pain is evaluated and if the given values reflect maximum or mean pain. When multiple VAS values are reported, high variations are observable.^22^^,^^29^^,^^37^^,^^40^

Concerning the occurrence of complications, our meta-analysis focused on the most severe and most consistently reported item, namely migration of the embolic agent or device. Our pooled data showed the highest rates of migration in coils; however, this effect can seemingly be augmented by the additional use of sclerosant. Coil migration is a well-known complication and has been described to have decreased in incidence over the last decade, from 4.2% to 1.6%,^44^ which did not consistently occur in our data. The successful snaring of coils is described by most authors; no fatal cases were described.^14^^,^^16^^,^^17^^,^^22^^,^^25^^,^^27^, ^28^, ^29^, ^30^ Additionally, the coil cohort had a 1.2% chance of the extravascular protrusion of coils, a specific complication of the method that may require surgical intervention.^45^ Cyanoacrylate glue migrated in a total of three cases (1.4%),^21^^,^^26^ leading to slight symptomatic pulmonary artery embolism in all the cases. Vascular plug pulmonary embolism occurred in two patients; it was asymptomatic in both patients and was only discovered through routine radiography.^16^^,^^17^ Only one case of migration of foam sclerosant was documented (0.5%).^40^ A contributing factor to the low number of events in the SG and GG might be their lower visibility in routine radiographs of the chest; asymptomatic pulmonary migration of sclerosant and glue could therefore remain undetected in many cases, potentially alluding to a significant dark number. Especially in the glue cohort, the mean follow-up time was markedly lower than in the other groups at only slightly more than 1 year. This could lead to the underestimation of complication, recurrence, and reintervention rates compared with the other groups. Sclerotherapy is distinctly associated with severe distal and proximal thrombosis, with rates of <0.1% to <1% and 0.01%, respectively, which was not described in any of the included studies.^44^ PES occurred in 4.5% of patients receiving coil embolization, 1.4% of patients receiving sclerosant, and in none of patients in the other groups. It was self-limiting under symptomatic treatment in all the instances.^25^^,^^37^^,^^38^

The sclerosant-only group had the least patients with recurrence across multiple studies with an above average follow-up time of almost 3 years. However, none of these studies reported reintervention. The one study that did report reintervention (but not recurrence) showed the highest number of reinterventions of all the studies at the longest mean follow-up interval of 99 months. Therefore, under-reporting may occur in the other studies, or a relevant portion of patients treated with sclerosant only show recurrence after multiple years.

Recurrence was only reported in 13 of 29 studies, heterogeneously defined and markedly higher in studies when radiological parameters were considered additionally to clinical parameters (12.4% vs 5.2%).

Vascular plugs alone showed very good results in all the outcome measures, with very good clinical success at the 12-month follow-up, as well as few events of complications, recurrence, and reintervention even when followed up >4 years in all the categories. The randomized controlled trial by Guirola et al^16^ additionally showed reduced intervention time as well as radiation dose, with only approximately 20% higher mean device cost. De Gregorio et al^18^ combined plugs with sclerosant; VAS score reduction was slightly higher than the average score across all the plug studies, and no device migration was observed. However, the cohort size was very limited.

One source of bias that could not be accounted for due to large variations between and even within protocols was the extent of treated vessels. Although most studies conducted limited embolization of only the venographically refluxing veins, some colleagues decided to standardize treatment by embolizing the ovarian and internal iliac veins bilaterally in all the patients. Liang et al^42^ recently attempted to analyze whether the extent of embolization affects the treatment efficacy in their retrospective study of 139 patients and found no significant differences in extensive vs more conservative embolization, but with high heterogeneity in the protocols. The Zaragoza group is currently the only group consistently opting for the occlusion of all the four veins.^16^, ^17^, ^18^ Across the coil cohorts in these studies, the reduction in VAS scores was similar to the group average in our meta-analysis; the clinical success rate was however higher than group average. The effect of the extent of embolization—especially on the long-term absence of symptoms—needs to be further elucidated in a standardized trial.

We chose a cut-off of >20 patients to ensure that only centers with sufficient expertise were included, so as to not skew results.

Regarding the limitations of this study, several points must be addressed. The performance of a network meta-analysis was not feasible due to the widely different interventional protocols and no standardized control groups. We therefore grouped individual treatment cohorts. Therefore, our data do not allow for between-group statistical analysis. All analyses were purely descriptive. In the analysis of clinical success, recurrence, and reintervention rates and especially in the statistical analysis of migration, CIs overlap considerably. Follow-up intervals varied greatly between the groups, but also within groups and individual studies (Table I). A substantial number of (retrospective) studies describe fixed follow-up dates (eg, at 1, 6, and 12 months) without describing whether these dates were closely adhered to. Especially, more established therapies such as coil-based procedures tended to have longer follow-ups than glue or sclerosant when analyzing recurrence, which could explain their seemingly worse performance.

As mentioned above, the extent of embolization was widely heterogeneous, with some studies even including patients treated for lower extremity or vulvar varicosis prior. The specific site of intervention complications (eg, protrusion, rupture, and PES) was recorded but not analyzed due to limited data. Nonintervention-specific complications such as access-site complications or adverse reactions to pain medication were not recorded. Overall, the number of events was very low. Patient numbers varied greatly between the treatment groups, with coil-based embolization being by far the most prevalent, limiting the statistical analysis of subgroups.

As described above, pain—even when measured with a standardized tool such as VAS—is a subjective parameter that could be influenced by the intrinsic characteristics of the patient population as well as the examiner.

Concerning the evaluation of clinical success, the subjective nature of the parameter is even more obvious, as individual definitions of what constitutes success as well as the durations of follow-up intervals differ greatly. Recurrence varied widely depending on the definition. Reinterventions were only reported by seven studies. Statistical analysis is therefore of very limited reliability. Additionally, the cause of reintervention is rarely mentioned and might include reintervention due to complications rather than due to recurrence or insufficient control of symptoms. Finally, follow-up times varied markedly between the intervention groups with ROB, especially with respect to recurrence and reinterventions.

## Conclusions

All the embolization methods analyzed are effective in reducing pain in patients with CPP due to PeVD and show overall low rates of severe adverse events. Most studies use coils alone or in combination with sclerosant. Device migration is mainly seen with the use of coils or glue. Larger and ideally randomized cohorts and standardized follow-up times are needed for more robust analysis.

## Author Contributions

Conception and design: FS, MB, JF, HJ, AG, LB

Analysis and interpretation: FS, AB

Data collection: FS, LM, LB

Writing the article: FS, AB, LM, LB

Critical revision of the article: FS, AB, LM, MB, JF, HJ, AG, LB

Final approval of the article: FS, AB, LM, MB, JF, HJ, AG, LB

Statistical analysis: FS, AB

Obtained funding: Not applicable

Overall responsibility: FS

## Declaration of generative AI and AI-assisted technologies in the writing process

No generative AI-tools were used in the writing of this manuscript. The authors take full responsibility for the content of the publication.

## Data availability

The datasets generated and analyzed during the current study are available from the corresponding author upon reasonable request.

## Funding

None.

## Disclosures

None.

## References

[1] (2020). Chronic pelvic pain: ACOG practice bulletin summary, number 218. Obstet Gynecol.

[2] Meissner M.H., Gibson K. (2015). Clinical outcome after treatment of pelvic congestion syndrome: sense and nonsense. Phlebology.

[3] Khilnani N.M., Meissner M.H., Learman L.A. (2019 Jun). Research priorities in pelvic venous disorders in women: recommendations from a multidisciplinary research consensus panel. J Vasc Interv Radiol.

[4] Meissner M.H., Khilnani N.M., Labropoulos N. (2021). The symptoms-varices-pathophysiology classification of pelvic venous disorders: a report of the American Vein & Lymphatic Society International Working Group on pelvic venous disorders. J Vasc Surg Venous Lymphat Disord.

[5] Jaworucka-Kaczorowska A., Roustazadeh R., Simka M., Jalaie H. (2025). Management of extra-pelvic varicose veins of pelvic origin in female patients. J Clin Med.

[6] Soysal M.E., Soysal S., Vicdan K., Ozer S. (2001). A randomized controlled trial of goserelin and medroxyprogesterone acetate in the treatment of pelvic congestion. Hum Reprod Oxf Engl.

[7] Clark M.R., Taylor A.C. (2023). Pelvic venous disorders: an update in terminology, diagnosis, and treatment. Semin Interv Radiol.

[8] Pennec V.L., Douane F., Brun J.L. (2025). Endovascular management of pelvic congestion syndrome: an expert consensus statement from the French Society of Cardiovascular Imaging (SFICV), Interventional Radiology Federation (FRI), College of French Radiology Teachers (CERF), and French Society of Women’s Imaging (SIFEM). Diagn Interv Imaging.

[9] Gavrilov S., Karalkin A., Mishakina N., Efremova O., Grishenkova A. (2022). Relationships of pelvic vein diameter and reflux with clinical manifestations of pelvic venous disorder. Diagnostics.

[10] Gavrilov S.G., Karalkin A.V., Mishakina N.Y., Grishenkova A.S. (2023). Hemodynamic and neurobiological factors for the development of chronic pelvic pain in patients with pelvic venous disorder. J Vasc Surg Venous Lymphat Disord.

[11] Krzanowski M., Partyka L., Drelicharz L. (2019). Posture commonly and considerably modifies stenosis of left common iliac and left renal veins in women diagnosed with pelvic venous disorder. J Vasc Surg Venous Lymphat Disord.

[12] Lakhanpal G., Kennedy R., Lakhanpal S., Sulakvelidze L., Pappas P.J. (2021). Pelvic venous insufficiency secondary to iliac vein stenosis and ovarian vein reflux treated with iliac vein stenting alone. J Vasc Surg Venous Lymphat Disord.

[13] Rossi F.H., Kambara A.M. (2026). Paradigm shift and long-term results in the diagnosis and treatment of pelvic venous disorder. J Vasc Surg Venous Lymphat Disord.

[14] Nasser F., Cavalcante R.N., Affonso B.B., Messina M.L., Carnevale F.C., de Gregorio M.A. (2014). Safety, efficacy, and prognostic factors in endovascular treatment of pelvic congestion syndrome. Int J Gynaecol Obstet.

[15] De Carvalho S.F.C., Metzger P.B., Fernandez M.G., Ribeiro W.B., Nogueira A.K.S., Souza J.P.R.E. (2023). Pelvic venous reflux embolization in the treatment of symptomatic pelvic congestive syndrome: a systematic review with meta-analysis. J Vasc Surg Venous Lymphat Disord.

[16] Guirola J.A., Sánchez-Ballestin M., Sierre S., Lahuerta C., Mayoral V., De Gregorio M.A. (2018). A randomized trial of endovascular embolization treatment in pelvic congestion syndrome: fibered platinum coils versus vascular plugs with 1-year clinical outcomes. J Vasc Interv Radiol.

[17] De Gregorio M.A., Guirola J.A., Alvarez-Arranz E., Sánchez-Ballestin M., Urbano J., Sierre S. (2020). Pelvic venous disorders in women due to pelvic varices: treatment by embolization: experience in 520 patients. J Vasc Interv Radiol.

[18] De Gregorio M.Á., Yamamoto-Ramos M., Fredes A. (2023). A comparative study of a small series of patients (50 patients) with pelvic varicose veins treated with plugs alone or plugs and polidocanol. J Clin Med.

[19] Chen H., Wu Z., Wu Z. (2023). Proximal coil occlusion preceding distal sclerotherapy in patients with pelvic congestion syndrome: a multicenter, retrospective study. J Vasc Surg Venous Lymphat Disord.

[20] Zhou Z., Yang M., Guo P. (2024). Effectiveness and safety of coils plus glue in slope embankment technology versus coils plus sclerosant in embolization therapy for reflux-type pelvic venous disorders. J Vasc Surg Venous Lymphat Disord.

[21] Zaghloul H., Nasser M., El-Mawgoud H.A., Ghoneim B., Ali M. (2025). Coil occlusion combined with sclerotherapy versus N-butyl-2-cyanoacrylate embolization in patients with pelvic venous disorders: a single-center retrospective study. J Vasc Surg Venous Lymphat Disord.

[22] Yeşiltaş M.A., Ketenciler S., Yücel C., Koyuncu A.O., Sayili U. (2025). Comparison of embolization using coil versus coil and ethylene vinyl alcohol copolymer in pelvic venous disorders. Ann Vasc Surg.

[23] Black C.M., Thorpe K., Venrbux A. (2010). Research reporting standards for endovascular treatment of pelvic venous insufficiency. J Vasc Interv Radiol.

[24] Kwon D., Reis I.M. (2015). Simulation-based estimation of mean and standard deviation for meta-analysis via Approximate Bayesian Computation (ABC). BMC Med Res Methodol.

[25] Gavrilov S.G., Sazhin A.V., Akhmetzianov R. (2023). Surgical and endovascular treatment of pelvic venous disorder: results of a multicentre retrospective cohort study. J Vasc Surg Venous Lymphat Disord.

[26] Maleux G., Stockx L., Wilms G., Marchal G. (2000). Ovarian vein embolization for the treatment of pelvic congestion syndrome: long-term technical and clinical results. J Vasc Interv Radiol.

[27] Venbrux A.C., Chang A.H., Kim H.S. (2002). Pelvic congestion syndrome (pelvic venous incompetence): impact of ovarian and internal iliac vein embolotherapy on menstrual cycle and chronic pelvic pain. J Vasc Interv Radiol.

[28] Chung M.H., Huh C.Y. (2003). Comparison of treatments for pelvic congestion syndrome. Tohoku J Exp Med.

[29] Kim H.S., Malhotra A.D., Rowe P.C., Lee J.M., Venbrux A.C. (2006). Embolotherapy for pelvic congestion syndrome: long-term results. J Vasc Interv Radiol.

[30] Kwon S.H., Oh J.H., Ko K.R., Park H.C., Huh J.Y. (2007). Transcatheter ovarian vein embolization using coils for the treatment of pelvic congestion syndrome. Cardiovasc Intervent Radiol.

[31] Gandini R., Chiocchi M., Konda D., Pampana E., Fabiano S., Simonetti G. (2008). Transcatheter foam sclerotherapy of symptomatic female varicocele with sodium-tetradecyl-sulfate foam. Cardiovasc Intervent Radiol.

[32] van der Vleuten C.J.M., van Kempen J.A.L., Schultze-Kool L.J. (2012). Embolization to treat pelvic congestion syndrome and vulval varicose veins. Int J Gynaecol Obstet.

[33] Laborda A., Medrano J., de Blas I., Urtiaga I., Carnevale F.C., de Gregorio M.A. (2013). Endovascular treatment of pelvic congestion syndrome: visual analog scale (VAS) long-term follow-up clinical evaluation in 202 patients. Cardiovasc Intervent Radiol.

[34] Gandini R., Konda D., Abrignani S. (2014). Treatment of symptomatic high-flow female varicoceles with stop-flow foam sclerotherapy. Cardiovasc Intervent Radiol.

[35] Edo Prades M.A., Ferrer Puchol M.D., Esteban Hernández E., Ferrero Asensi M. (2014). [Pelvic congestion syndrome: outcome after embolization with coils]. Radiologia.

[36] Siqueira F.M., Monsignore L.M., Rosa-E-Silva J.C. (2016). Evaluation of embolization for periuterine varices involving chronic pelvic pain secondary to pelvic congestion syndrome. Clin Sao Paulo Braz.

[37] Gavrilov S.G., Sazhin A., Krasavin G., Moskalenko E., Mishakina N. (2021). Comparative analysis of the efficacy and safety of endovascular and endoscopic interventions on the gonadal veins in the treatment of pelvic congestion syndrome. J Vasc Surg Venous Lymphat Disord.

[38] Jambon E., Le Bras Y., Coussy A. (2022). Embolization in pelvic venous disorders using ethylene vinyl alcohol copolymer (Onyx®) and Aetoxysclerol: a prospective evaluation of safety and long-term efficacy. Eur Radiol.

[39] Ignacio Leal Lorenzo J., Gallardo Madueño G., Alcázar Peral A., Pillado Rodríguez E., Cárdenas Santos R., Alonso Burgos A. (2022). Bilateral ovarian vein embolisation from a unilateral basilic approach with n-2-Butyl cyanoacrylate and crossover technique for pelvic congestion syndrome. Eur J Vasc Endovasc Surg.

[40] Shahat M., Hussein R.S., Ahmed A.K.S. (2023). Foam sclerotherapy in pelvic congestion syndrome. Vasc Endovascular Surg.

[41] Liang E., Wong W.Y.T., Parvez R., Chan M., Brown B. (2025). A keep-it-simple embolisation approach to treat pelvic congestion syndrome without compromising clinical effectiveness. J Med Imaging Radiat Oncol.

[42] Wang P., Zheng X.L., Wang Y.H., Tao Y., Wang W.C. (2025). Analysis of the clinical efficacy of interlock detachable coil interventional embolization on pelvic congestion syndrome and ovarian reserve function: a retrospective study. Int J Gen Med.

[43] Hipola J.M., Alonso A., Cárdenas R., Pillado E., Leal J.I. (2025). Efficacy and safety of ovarian vein embolization with N-butyl-2 cyanoacrylate for pelvic venous disorder: analysis of 100 cases. J Vasc Surg Venous Lymphat Disord.

[44] Lazarashvili Z., Hirsch T. (2021). Complications and side effects after pelvic vein embolization. Turk J Vasc Surg.

[45] Gavrilov S.G., Mishakina N.Y., Efremova O.I., Kirsanov K.V. (2022). Complications and adverse events of gonadal Vein embolization with coils. J Pers Med.

