# Supplementary material for: Pelvic vein embolization using different embolic agents for the treatment of pelvic venous insufficiency: A systematic review and meta-analysis
Source: J Vasc Surg Venous Lymphat Disord. 2026 Aug 9;14(6):102599. doi: 10.1016/j.jvsv.2026.102599 (PMC13590090; doi:10.1016/j.jvsv.2026.102599)
Supplement: Supplementary file 1 [file mmc1.docx]

Supplementary Data

**Supplementary Figure 1 – Risk of Bias Analysis** using the Risk of Bias In Non-randomized Studies – of Interventions (ROBINS-I) tool

**Supplementary Figure 2 – Risk of Bias Analysis** using the Risk-of-bias Tool for Randomized Trials (ROB-2)

Supplementary Data 1: Search Terms and Yields

Searched on MEDLINE on 24/10/2025

"pelvic congestion syndrome" OR "pelvic venous disease*" OR "pelvic venous disord*" OR "pelvic venous insufficiency"

Results 525

- Fitting results 54 (intervention studies on PeVD)
- only pharmacological treatment 4
- only venous obstruction, not insufficiency 8
- studies on embolization therapy 42
  - ≤ 20 patients 10
  - studies with inconsistent protocols 5
  - Included 27 studiesChCh

Search on Science Direct on 28/11/2025

"pelvic congestion syndrome" OR "pelvic venous disease" OR "pelvic venous disorder" OR "pelvic venous insufficiency"

Results 1,236

- Research Articles and Short Communications 414
- Fitting results 37
  - Only pharmacological 2
  - Only venous obstruction 12
  - Embolization 23
    - Duplicates 21
    - No outcome parameters of interest reported 1
    - Included 1

"pelvic congestion syndrome" OR "pelvic venous disease*" OR "pelvic venous disord*" OR "pelvic venous insufficiency"

Searched on ClinicalTrials.gov on 28/11/2025

Results 133

- Incomplete 80
- Completed 53
  - Fitting Results 1
    - Duplicate 1

"pelvic congestion syndrome" OR "pelvic venous disease" OR "pelvic venous disorder" OR "pelvic venous insufficiency"

Searched on Cochrane Central Register of Controlled Trials on 28/11/2025

Results 33

- Fitting Results 5
  - No Full Text 2
  - Duplicates
